# Response to Comments on ‘Hepatitis B Virus (HBV) Treatment Eligibility in the UK: Retrospective Longitudinal Cohort Data to Explore the Impact of Changes in Clinical Guidelines’

**DOI:** 10.1111/jvh.70133

**Published:** 2026-01-23

**Authors:** Cori Campbell, Tingyan Wang, Eleanor Barnes, Philippa C. Matthews, the UK Health Informatics Collaborative for Viral Hepatitis and Liver Disease

**Affiliations:** ^1^ NIHR Oxford Biomedical Research Centre Oxford UK; ^2^ Nuffield Department of Medicine University of Oxford Oxford UK; ^3^ NIHR Health Informatics Collaborative Oxford University Hospitals NHS Foundation Trust Oxford UK; ^4^ Department of Hepatology Oxford University Hospitals NHS Trust. Oxford UK; ^5^ The Francis Crick Institute London UK; ^6^ Division of Infection and Immunity University College London London UK; ^7^ Department of Infectious Diseases University College London Hospital London UK

Dear Editor,

Thank you for the opportunity to respond to the letter from Katkuri et al. [1]. We thank this group for providing positive feedback on our report of treatment eligibility for adults living with Hepatitis B virus (HBV) infection in the UK [2], which was undertaken by the Health Informatics Collaborative (HIC) for viral hepatitis and liver disease [3].

First, we address the feedback about the HIC approach of using a single positive HBV test as a marker of chronic infection. We recognise that this approach deviates from the gold standard of two tests over a period of at least 6 months, and may therefore (in theory) misassign some cases of acute infection as chronic. However, the decision to adopt this relaxed approach to case definition was taken and ratified by an expert board with clinical and data experts from all the HIC sites and has been previously justified, published and peer reviewed [4]. In England, the incidence of acute HBV infection has been declining over time, and is low (< 300 cases/year since 2020 [5]). The potential for acute infection to influence any analytical conclusions based on analysis of the population under chronic care clinics is therefore negligible. Based on the longitudinal follow‐up in this dataset, the chances of including acute cases is further reduced (those with acute infection will not have data points for inclusion beyond 6 months under observation). By removing those with only one diagnostic test, we would risk excluding valuable data from people with true chronic infection.

Second, in considering our statistical methods and presentation of results, we agree there is a potential problem of reverse causality. On these grounds we analysed data and presented conclusions carefully, deliberately avoiding characterisation of any associations as causal. On these grounds, we simply present ‘factors associated with treatment’ without any statements on either temporality or causality, which we are indeed unable to assign in this analysis. Note that figure 1 in our paper [2] presents an odds ratio for ‘ever receiving’ treatment which does not imply any direction of association.

Many factors associated with treatment status (such as sex, chronic co‐infection, ethnicity, and IMD quintile) are not subject to temporality. However, we recognise that the lack of significant association between viral load and treatment is likely explained by a temporality issue (viral load is lower in those receiving treatment and therefore—despite being a major part of eligibility assessment—does not emerge as a significant signal predicting treatment. This point is addressed in the paper's discussion [2]). We agree and acknowledge that there are limitations associated with gaps in recording of treatment start date; this is explicitly included in the original paper (section 4.5 in the discussion expands on this challenge in some detail).

Third, in terms of appraising treatment eligibility based on HIC data, it is indeed the case that the approach taken is simplified. We explain why in the paper: this national collaborative process captures demographic, imaging and laboratory data, as quantitative variables are robustly captured using electronic systems [2]. Other variables such as family history, comorbidity, or personal preference are routinely applied to decision‐making in real‐world clinical practice but are unfortunately not yet consistently captured by electronic records. Our section entitled ‘caveats and limitations’ describes this issue, and we go on to elaborate on the implications of these missing parameters, which could lead to either under‐ or over‐estimation of the proportion who would receive treatment following personalised assessment. Katkuri and co‐authors propose a sensitivity analysis as an approach to investigating the impact of these missing data. However, such an undertaking cannot be applied when these parameters are simply not collected across the whole dataset (a sensitivity analysis would only have been possible if there were missing data for a subset of the cohort, which could be defined and removed for a more granular re‐analysis).

We have further explored approaches to missing parameters in our paper's section on future aspirations, in which the need for enhanced data is described. Some of these parameters, such as ICD‐10 codes for diagnosis, will allow future expansion. Other data, such as personal preferences, remain less easy to document on a population level, as this is a nuanced discussion between a patient and their care provider which is not captured on existing electronic platforms. We clearly acknowledge these caveats, and for this reason present our work as a ‘preliminary’ descriptive analysis.

Finally, Katkuri highlights small numerical errors. We aspire to meticulous and error‐free reporting, and therefore regret that two mistakes in section 3.3 were not spotted ahead of publication, as follows:

- Category (iii): Applying the WHO treatment criterion of two ALT measurements exceeding the upper limit of normal during any 6–12‐month period, the proportion of individuals meeting eligibility under this scenario stated in the article is correctly presented as 2740/7558, but the percentage should be 36.3% (not 32.3% as stated)
- Category (ix) has an error in the denominator, which is reported as 7887 (instead of 7558), so the calculation here should be 7178/7558 (the proportion should be 94.9%, rather than 95.1% as stated).

We agree with Katkuri's point that these discrepancies are numerically small, and do not alter the magnitude or trend in treatment outcomes, nor do they affect qualitative conclusions (on these grounds, the journal editorial team has advised that publication of a formal Erratum is not required).

Overall, we are grateful for the thoughtful and detailed feedback on our publication. This dialogue is an interesting opportunity to reflect on, discuss and learn from the opportunities and challenges of using real‐world clinical data. Despite the caveats, our reported analysis is a stepping‐stone to establish patterns that are relevant to planning policy and services for tackling HBV at a population level.

As a clinical and research community, we must undoubtedly continue to advance and improve the ways in which routine health service data are confidentially collated and interrogated to understand population‐level characteristics and needs [6]. These aspirations are highlighted as part of National Health Service strategy for the UK, as part of a drive to move from analogue to digital healthcare [7]. As the global community works towards wider HBV treatment roll‐out as a component of strategies that will advance us towards elimination of viral hepatitis as a public health threat by 2030 [8], ongoing investment in access to high‐quality population data is an essential aspiration.

## Funding

P.C.M. is supported by the Francis Crick Institute which receives its core funding from Cancer Research UK, the UK Medical Research Council, and the Wellcome Trust (ref CC2223). She also receives funding from the University College London Hospitals NIHR Biomedical Research Centre (BRC).

## Conflicts of Interest

C.C. has received funding support from GSK towards her PhD fellowship, supervised by P.C.M. and E.B. (2019–2023). The UK Health Informatics Collaborative for viral hepatitis and liver disease acknowledges funding from NIHR and GSK. P.C.M. is co‐chair of the UK National Strategic Group for Viral Hepatitis (NSGVH), receives royalties from Oxford University Press for book publications, and has received speaker fees from J&J for an educational event (2025). E.B. holds patents in HBV vaccines and has received funding for research from GSK and Barinthus in the field of HBV.

## Linked Article

Comments on “Hepatitis B Virus (HBV) Treatment Eligibility in the UK: Retrospective Longitudinal Cohort Data to Explore the Impact of Changes in Clinical Guidelines” https://doi.org/10.1111/jvh.70132.

## Data Availability

Data sharing not applicable to this article as no datasets were generated or analysed during the current study.

## References

[1] S. N. Katkuri , V. Vadhithala , A. Kumar , S. Verma , and D. Dedeepyaa , “Comments on ‘Hepatitis B Virus (HBV) Treatment Eligibility in the UK: Retrospective Longitudinal Cohort Data to Explore the Impact of Changes in Clinical Guidelines’,” Journal of Viral Hepatitis 33 (2025): e70132. 10.1111/jvh.70132 41508996

[2] C. Campbell , T. Wang , A. J. Stockdale , et al., “Hepatitis B Virus (HBV) Treatment Eligibility in the UK: Retrospective Longitudinal Cohort Data to Explore the Impact of Changes in Clinical Guidelines,” Journal of Viral Hepatitis 32 (2025): e70098. 41128166 10.1111/jvh.70098 PMC12548009

[3] NIHR , “Viral Hepatitis and Liver Disease,” accessed December 22, 2025, https://hic.nihr.ac.uk/viral‐hepatitis.

[4] T. Wang , D. A. Smith , C. Campbell , et al., “Cohort Profile: The National Institute for Health Research Health Informatics Collaborative: Hepatitis B Virus (NIHR HIC HBV) Research Dataset,” International Journal of Epidemiology 52 (2023): e27–e37. 35708657 10.1093/ije/dyac127 PMC9908046

[5] GOV. UK , “Hepatitis B in England 2025, GOV.UK,” accessed December 22, 2025, https://www.gov.uk/government/publications/hepatitis‐b‐in‐england/hepatitis‐b‐in‐england‐2025.

[6] C. Sudlow , “The Sudlow Review, HDR UK [Internet]” accessed December 22, 2025, https://www.hdruk.ac.uk/helping‐with‐health‐data/the‐sudlow‐review/.

[7] GOV.UK , “Road to Recovery: The Government's 2025 Mandate to NHS England,” accessed December 22, 2025, https://www.gov.uk/government/publications/road‐to‐recovery‐the‐governments‐2025‐mandate‐to‐nhs‐england/road‐to‐recovery‐the‐governments‐2025‐mandate‐to‐nhs‐england.

[8] WHO , “Global Health Sector Strategies,” accessed December 22, 2025, https://www.who.int/teams/global‐hiv‐hepatitis‐and‐stis‐programmes/strategies/global‐health‐sector‐strategies.

